# The SKP1-Cullin-F-box E3 ligase βTrCP and CDK2 cooperate to control STIL abundance and centriole number

**DOI:** 10.1098/rsob.170253

**Published:** 2018-02-14

**Authors:** Christian Arquint, Fabien Cubizolles, Agathe Morand, Alexander Schmidt, Erich A. Nigg

**Affiliations:** Biozentrum, University of Basel, Klingelbergstrasse 50/70, 4056 Basel, Switzerland

**Keywords:** SCF-βTrCP, STIL, CDK2, centriole duplication, centrosome, centriole number

## Abstract

Deregulation of centriole duplication has been implicated in cancer and primary microcephaly. Accordingly, it is important to understand how key centriole duplication factors are regulated. E3 ubiquitin ligases have been implicated in controlling the levels of several duplication factors, including PLK4, STIL and SAS-6, but the precise mechanisms ensuring centriole homeostasis remain to be fully understood. Here, we have combined proteomics approaches with the use of MLN4924, a generic inhibitor of SCF E3 ubiquitin ligases, to monitor changes in the cellular abundance of centriole duplication factors. We identified human STIL as a novel substrate of SCF-βTrCP. The binding of βTrCP depends on a DSG motif within STIL, and serine 395 within this motif is phosphorylated *in vivo*. SCF-βTrCP-mediated degradation of STIL occurs throughout interphase and mutations in the DSG motif causes massive centrosome amplification, attesting to the physiological importance of the pathway. We also uncover a connection between this new pathway and CDK2, whose role in centriole biogenesis remains poorly understood. We show that CDK2 activity protects STIL against SCF-βTrCP-mediated degradation, indicating that CDK2 and SCF-βTrCP cooperate via STIL to control centriole biogenesis.

## 1. Introduction

In animal cells, centrosomes serve as microtubule organizing centres to shape the architecture of microtubule networks [1,2]. Accordingly, they influence microtubule-dependent processes, notably cell shape, polarity and motility [3]. Centrosomes also associate with mitotic spindle poles, and thereby participate in spindle formation and orientation, ensuring equal distribution of chromosomes during cell division [4,5]. Moreover, centrosomes may function as solid-state platforms to help accumulate proteins implicated in signalling processes [6]. Centrosomes comprise centrioles that are surrounded by a protein matrix, the pericentriolar material (PCM). While the PCM contains a multitude of proteins, including factors that nucleate and anchor microtubules, centrioles are composed of stabilized microtubule triplets, resulting in cylindrical structures with evolutionary conserved ninefold symmetry [7,8]. Centrioles not only form the core of centrosomes, but also function as basal bodies to allow ciliogenesis [9,10]. In quiescent cells, docking of a centriole to the plasma membrane promotes the outgrowth of a primary cilium, which functions as an antenna to receive and integrate extracellular stimuli [11,12].

In cycling cells, centrioles are duplicated exactly once during S phase [13–15]. While G1 phase cells contain one centrosome comprising two loosely connected centrioles, at the G1/S phase transition, one new procentriole is assembled in orthogonal orientation near the proximal end of each pre-existing (parental) centriole. The newly formed procentrioles then elongate throughout G2 phase, but initially remain unable to recruit PCM [16,17]. Procentrioles remain closely connected to their parents, until centriole disengagement is triggered during mitosis by Separase and PLK1 activities [4,18]. Also at mitotic entry, the flexible protein linker connecting parental centrioles is dissolved [19] and the PCM enlarges in a process termed centrosome maturation [8]. This results in the formation of two independent centrosomes with high microtubule nucleation capacity, ready to participate in bipolar spindle formation.

Centriole duplication requires the sequential interaction between a defined set of centriole duplication factors. During initial steps, PLK4, STIL and SAS-6 cooperate in the assembly of a ninefold symmetrical structure, termed cartwheel, that then supports procentriole formation [7,14,20]. To trigger the process, the kinase PLK4 is recruited by CEP152 and CEP192 to the proximal base of each parental centriole [21–23]. Then, towards the G1/S transition, STIL and SAS-6 co-localize with PLK4 to the future site of procentriole formation [23–26]. The exact mechanisms that determine the location of procentriole formation on the circumference of procentrioles remain to be understood, but the available evidence indicates that PLK4 first binds to STIL and phosphorylates STIL's STAN domain, promoting STIL interaction with SAS-6 [25–29]. In addition, the STIL–PLK4 interaction has been demonstrated to increase PLK4's kinase activity, which in turn is likely to influence PLK4 abundance and localization [27,28]. Subsequently, other centriole duplication factors, notably CEP135, CPAP and CP110, are recruited to allow the building of the emerging procentriole and its elongation [15].

Deregulation of centriole duplication has been linked to a number of diseases, emphasizing the need for a better understanding of the mechanisms that regulate centriole numbers under physiological and patho-physiological conditions. Both numerical and structural centrosome aberrations are common in cancer cells, and have been linked to genome instability and invasion [30–37], as well as impaired ciliary signalling [38]. Furthermore, deregulation of centriole duplication has been implicated in the aetiology of primary microcephaly or dwarfism [39–43].

To prevent unscheduled formation of extra centrioles, regulatory mechanisms must exist to limit centriole duplication to once per cell cycle (cell cycle control) and to restrict the formation of procentrioles to one per parental centriole (copy number control) [44], and recent studies begin to elucidate the underlying mechanisms [15]. In brief, the licensing of centrioles for a new round of duplication (cell cycle control) is recognized to require centriole disengagement as well as centriole-to-centrosome conversion during M phase progression [16–18,45–48]. Regarding the mechanisms ensuring the formation of a single procentriole per pre-existing centriole (copy number control), most attention is focused on the question of how cells control PLK4 activity in time and space. Overexpression of PLK4, STIL or SAS-6 overrides copy number control and results in the near-simultaneous formation of multiple procentrioles [49–56], demonstrating that the abundance of these three centriole duplication factors and/or their recruitment to centrioles are tightly regulated.

Of particular interest, PLK4, STIL and SAS-6 are all regulated by proteasomal degradation [57]. This focused attention on two prominent cell cycle regulatory E3 ubiquitin ligases, the anaphase-promoting complex/cyclosome (APC/C) and the SKP1-CUL1-F-box-protein (SCF) ubiquitin ligase. While APC/C operates during mitosis and G1 phase, and uses the co-activators CDC20 and CDH1 for substrate recognition, SCFs operate throughout the cell cycle and require a large set of different F-box proteins for substrate recognition [58]. CDC20, CDH1 and F-box proteins all recognize short motifs, referred to as destruction motifs or degrons, on their substrate proteins, and most F-box proteins additionally require phosphorylation of the motifs [59]. SAS-6, STIL as well as CPAP all contain a KEN box destruction motif, which results in APC/C-CDH1-mediated degradation during late mitosis and early G1 phase [39,52,54,56,60]. This probably contributes to prevent unscheduled initiation of centriole duplication prior to the G1/S phase transition, when APC/C is inactivated through Emi1 binding and CDK2 activity [61]. Additionally, an auto-regulatory feedback mechanism prevents active PLK4 from reaching excessive levels that would otherwise trigger centriole overduplication: PLK4 trans-auto-phosphorylates on a DSG motif, which results in binding of the F-box protein βTrCP, followed by ubiquitination and degradation via the SCF-βTrCP pathway [62–65]. Finally, other F-box proteins have also been implicated in the regulation of centriole duplication. First, SAS-6 has been reported to be degraded not only by APC/C but also by SCF-FBXW5, leading to the proposal that inhibition of SCF-FBXW5 by PLK4-dependent phosphorylation during S phase triggers SAS-6 stabilization and cartwheel formation [66]. Second, the abundance of CP110 during G2 phase has been reported to be regulated by SCF-Cyclin F [67], in a process counteracted by the deubiquitinase USP33 [68].

To preserve genome stability, proliferating cells need to coordinate centriole duplication with DNA replication. While little is presently known about the mechanisms underlying this coordination, several studies point to a central role of the cell cycle regulatory kinase CDK2 [69–72]. This raises the question of how CDK2 contributes to control centriole duplication. CDK2 is known to phosphorylate CP110, but the consequences of this phosphorylation are not fully understood [73]. Likewise, the CDK2 substrates MPS1 and nucleophosmin have been reported to associate with centrosomes [74–76], but the role of these proteins in centriole duplication remains controversial. Thus, the role of CDK2 in centriole duplication remains largely enigmatic.

Here, we have sought to identify novel pathways contributing to the regulation of centriole biogenesis. We identify STIL as a novel substrate of the ubiquitin ligase SCF-βTrCP, and we present data to suggest that CDK2 controls both STIL abundance and localization.

## 2. Results

### 2.1. Inhibition of SCF E3 ubiquitin ligase complexes by MLN4924 triggers accumulation of STIL

Cellular levels of PLK4, the master regulator of centriole duplication, are tightly controlled by proteolysis in response to SCF-mediated PLK4 ubiquitination [62–65,77]. Furthermore, both SAS-6 and CP110 have been identified as substrates of SCF [66,67]. Motivated by these findings, we sought to identify novel substrates of SCF ubiquitin ligases, whose proteolytic degradation might be important for centriole homeostasis. To this end, we quantified cellular levels of candidate proteins by combining mass spectrometry approaches with the use of MLN4924, a small molecule that blocks all SCF-mediated protein degradation through inhibition of the NEDD8-activating enzyme.

In pilot experiments, we treated U2OS cells for 24 h with different concentrations of MLN4924, or DMSO for control, with the expectation that inhibition of SCF-βTrCP should result in PLK4 accumulation and, consequently, centriole amplification. Indeed, treatment of U2OS cells with 0.1 µM MLN4924 resulted in 25% of cells with more than four centrioles, including a population that showed near-simultaneous formation of multiple daughter centrioles (referred to as a flower-like arrangement; electronic supplementary material, figure S1*a*,*b*). Upon use of MLN4924 at 0.5 µM, the proportion of cells harbouring more than four centrioles was increased to greater than 60%, including 20% with flower-like arrangements (electronic supplementary material, figure S1*a*,*b*). Given that these latter conditions caused sufficient inhibition of SCF E3 ligases to trigger extensive centriole amplification, they were adopted for proteomics studies on HEK 293T cells. Sufficiently abundant cellular proteins could readily be quantified through non-targeted mass spectrometry. Additionally, low-abundance centrosomal proteins were quantified through application of a targeted approach based on parallel reaction monitoring (PRM). Making use of isotope-labelled reference peptides, PRM enables quantification of proteins that would be difficult to monitor by conventional mass spectrometry [78].

**Figure 1.**
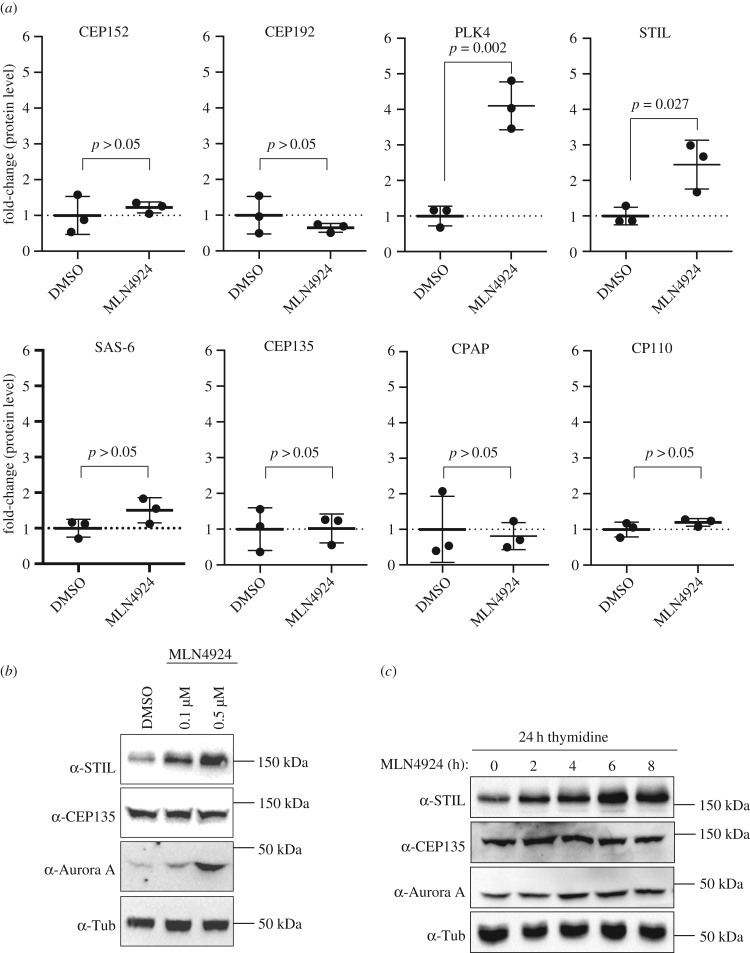
(*a*) Scatter plots show relative protein levels of the indicated centriole duplication factors in HEK 293T cells, as determined by PRM mass spectrometry. Cells were treated with either DMSO (control, *n* = 3) or 0.5 µM MLN4924 (*n* = 3) for 24 h. To indicate fold-changes in protein levels in response to MLN4924-treatment, the average values measured in DMSO-treated control cells were set to 1.0. Error bars denote s.d., *p*-values from *t*-tests (two-tailed, unpaired) are indicated. (*b*) Western blot analysis shows levels of STIL, CEP135 and Aurora A after 24 h treatment of HEK 293T cells with DMSO (control) or the indicated concentrations of MLN4924. Levels of α-tubulin were analysed for loading control. (*c*) Western blot analysis shows levels of STIL, CEP135 and Aurora A after treatment of HEK 293T cells with MLN4924 (0.5 µM) for the indicated times (h). Prior to drug addition, cells had been synchronized by a 24 h thymidine treatment. Levels of α-tubulin were analysed for loading control.

To demonstrate the sensitivity and reliability of our proteomics approaches, we first measured the effects of SCF inhibition on several abundant cellular proteins, including three well-established substrates of SCF E3 ubiquitin ligases, Aurora A [79,80], β-Catenin [81] and ORC1 [82] (electronic supplementary material, figure S1*c*). While the levels of these known SCF substrates markedly increased upon MLN4924 treatment, those of housekeeping proteins, notably tubulin α, tubulin β and glyceraldehyde-3-phosphate dehydrogenase (GAPDH) were not altered, as expected (electronic supplementary material, figure S1*d*). Following this validation, we next used PRM to measure the effects of SCF inhibition on the levels of eight proteins previously implicated in centriole duplication, notably PLK4, CEP152, CEP192, SAS-6, CEP135, CPAP, CP110 and STIL (figure 1*a*). As expected, PLK4, a well-established substrate of SCF-βTrCP ubiquitination [62–65,77], showed a strong accumulation upon MLN4924 treatment. Furthermore, and most intriguingly, we also observed a robust accumulation of the centriole duplication factor STIL. By contrast, no significant accumulations were seen for CEP152, CEP192, SAS-6, CEP135, CPAP and CP110. Considering that SAS-6 and CP110 have previously been reported to constitute targets of SCF-FBW5 [66] and SCF-Cyclin F [67], respectively, we were surprised that these proteins showed no significant accumulations. One possible explanation is that SAS-6 and CP110 might be recognized by their respective F-box proteins only during specific cell cycle stages, which might make it difficult to detect their stabilization in an experiment performed on asynchronously growing cells.

STIL is known to be degraded by APC/C-CDH1 during late M and early G1 phase [39,54,56,83]. However, an involvement of an SCF ubiquitin ligase in STIL regulation has not previously been reported. This prompted us to corroborate our mass spectrometry data by treating HEK 293T cells with MLN4924 and then monitoring STIL protein levels by western blotting (figure 1*b*). Already at 0.1 µM MLN4924, accumulation of STIL could readily be detected, and a further increase was seen at 0.5 µM. CEP135, analysed as negative control, showed no increase, whereas Aurora A, examined as positive control, strongly accumulated with increasing doses of MLN4924. These results fully corroborate the mass spectrometry data shown in figure 1*a*.

Given that MLN4924 has been reported to induce a G2 arrest in certain cell types [84] and that STIL protein levels increase towards G2 phase of the cell cycle [54,56], it was important to exclude that the observed accumulation of STIL might simply reflect a G2 arrest. To this end, the mass spectrometry-based quantification experiments were repeated in cells that had been thymidine-arrested in S phase prior to MLN4924 treatment. As seen for asynchronously growing cells, we again observed a significant upregulation for both PLK4 and STIL, demonstrating that accumulation of STIL cannot be attributed to a G2 cell cycle arrest (electronic supplementary material, figure S1*e*). Furthermore, we detected a minor upregulation of SAS-6, in line with the proposed SCF-mediated turnover in S-phase-arrested cells [66]. To assess the kinetics of STIL accumulation, we next conducted a time course experiment in S-phase-arrested HEK 293T cells and used western blotting to monitor STIL levels every 2 h, for a total of 8 h, after MLN4924 addition (figure 1*c*). An increase in STIL could be detected within a few hours of drug addition. Accumulation of Aurora A showed a comparable response, while CEP135 levels remained constant throughout the time of treatment, as expected. These results confirm that the observed accumulation of STIL is not due to perturbation of cell cycle progression.

Importantly, massive centriole amplification induced by PLK4 overexpression did not result in increased cellular STIL abundance (electronic supplementary material, figure S2), indicating that the MLN4924-induced STIL stabilization is not an indirect consequence of centriole amplification and/or PLK4 overexpression. Instead, our data raise the possibility that STIL is a direct target of an SCF E3 ubiquitin ligase.

### 2.2. βTrCP recognizes a conserved but non-canonical DSG motif within STIL

**Figure 2.**
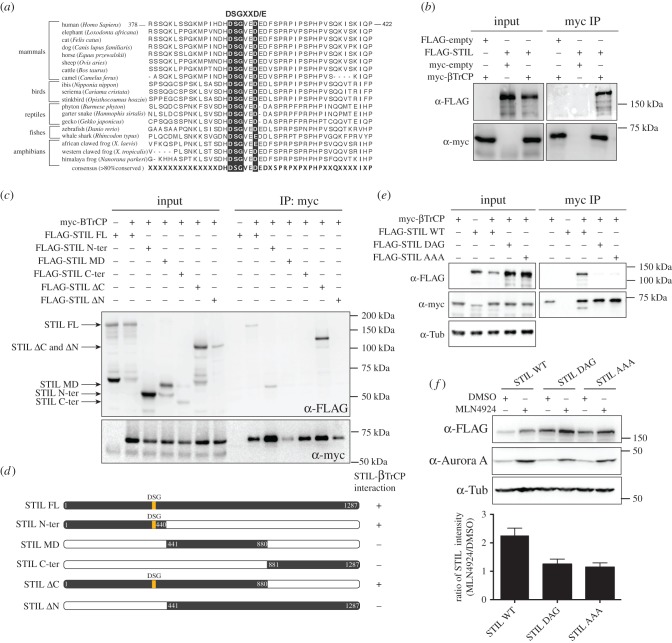
(*a*) Multiple protein sequence alignment (ClustalW) of vertebrate STIL sequences. The consensus sequence (more than 80% conserved) is shown at the bottom and taxonomic classes are shown on the side. The region encompassing the DSG motif is highlighted with black boxes. (*b*) HEK 293T cells overexpressing FLAG-STIL and myc-βTrCP for 36 h were lysed and subjected to immunoprecipitation (IP) using anti-myc antibodies. Bound proteins were separated by SDS–PAGE and analysed by western blotting using antibodies against the FLAG and myc tags. (*c*) The same experimental set-up as in (*b*), except that different FLAG-STIL truncations (depicted in (*d*)) were co-expressed with myc-βTrCP. (*d*) Schematic depicting the structures of FLAG-STIL WT and FLAG-STIL truncations used in (*c*). (*e*) Same experimental set-up as in (*b*), except that FLAG-STIL versions carrying mutations in the DSG motif (DAG, AAA) were co-expressed with myc-βTrCP. (*f*) HEK 293T cells were transfected with either FLAG-STIL WT, FLAG-STIL DAG or FLAG-STIL AAA for 36 h and subsequently treated with either DMSO (control) or MLN4924 for 24 h. Cell lysates were separated by SDS–PAGE and analysed by western blotting using antibodies against the FLAG tag and Aurora A (positive control illustrating effect of MLN4924 treatment). α-tubulin was analysed as loading control. The histogram at the bottom depicts the ratios (MLN4924 treatment relative to DMSO) of STIL WT, STIL DAG and STIL AAA levels, as measured by western blotting in three independent experiments.

SCF E3 ligases comprise F-box proteins that recognize well-defined amino acid sequences, so-called degron motifs, within substrate proteins [59]. Inspection of the human STIL amino acid sequence for the presence of potential binding sites for F-box proteins revealed a DSG motif within the N-terminal part (residues 394–399) (figure 2*a*). However, while canonical DSG motifs usually contain two phospho-acceptor sites (DSGXXS) that permit βTrCP binding in response to double phosphorylation, in the putative DSG motif of STIL, the second phosphorylation site has been replaced by aspartate (DSGXXD). This is not *a priori* expected to prevent degron recognition by βTrCP, as illustrated by the case of CDC25 phosphatases, which undergo destruction through recognition of a motif in which both phosphorylation sites are replaced by aspartate (DDGXXD) [85]. The DSG motif within STIL is well conserved among vertebrates (figure 2*a*), except for many rodents (electronic supplementary material, figure S3), but not in ANA2 or SAS-5, the STIL orthologues in *Drosophila melanogaster* and *Caenorhabditis elegans*, respectively. Importantly, S395 within DSG is one of the most prevalent phosphorylation sites within STIL listed on www.phosphosite.org, and the site can readily be phosphorylated *in vivo*, as indicated by mass spectrometry experiments detecting the corresponding phosphopeptide in overexpressed STIL (electronic supplementary material, figure S4).

To directly test whether βTrCP can bind STIL, lysates from transfected HEK 293T cells were used for co-immunoprecipitation experiments. Full-length FLAG-STIL could readily be co-immunoprecipitated with myc-βTrCP immobilized on beads (figure 2*b*). Furthermore, analysis of different truncated versions of STIL confirmed that STIL binding to myc-βTrCP required the presence of the DSG motif within N-terminal fragments (figure 2*c*,*d*), and mutation of the central serine residue to alanine (DAG) or replacement of all three amino acids to alanine (AAA) abrogated βTrCP binding (figure 2*e*). Given that S395 is a major STIL phosphorylation site *in vivo*, this strongly suggests that phosphorylation of the DSG motif contributes to strengthen STIL–βTrCP interaction, but because replacement of S395 by aspartate also abrogated STIL binding, this phospho-mimetic mutant could not be used to probe the effects of constitutive STIL phosphorylation. Taken together, the above data corroborate the conclusion that interactions between STIL and βTrCP occur *in vivo*. Further evidence supporting an interaction between STIL and βTrCP within cells stems from a large-scale Bio-ID proximity labelling screen: use of STIL as a bait led to the identification of both βTrCP paralogues, βTrCP1 (FBXW1) and βTrCP2 (FBXW11), as well as the SCF core component Cul1 [86], indicating that all these proteins are interacting with each other, or at least in close proximity.

The above data raised the question of whether the MLN4924-induced accumulation in STIL could be attributed exclusively to SCF-βTrCP acting on the DSG motif, or whether additional MLN4924-sensitive pathways might also be involved. To address this issue, we analysed the effect of MLN4924 treatment on STIL mutants with impaired DSG functionality (figure 2*f*). Compared with transfected STIL WT, which was present at low levels but accumulated strongly in response to MLN4924, both STIL DAG and STIL AAA were expressed at higher levels, and only minor additional accumulation could be detected in response to the drug. These results indicate that accumulation of STIL in response to MLN4924 treatment results primarily, but not exclusively, from inhibition of STIL degradation mediated by SCF-βTrCP acting through the DSG motif.

### 2.3. Cytoplasmic STIL is degraded in interphase

**Figure 3.**
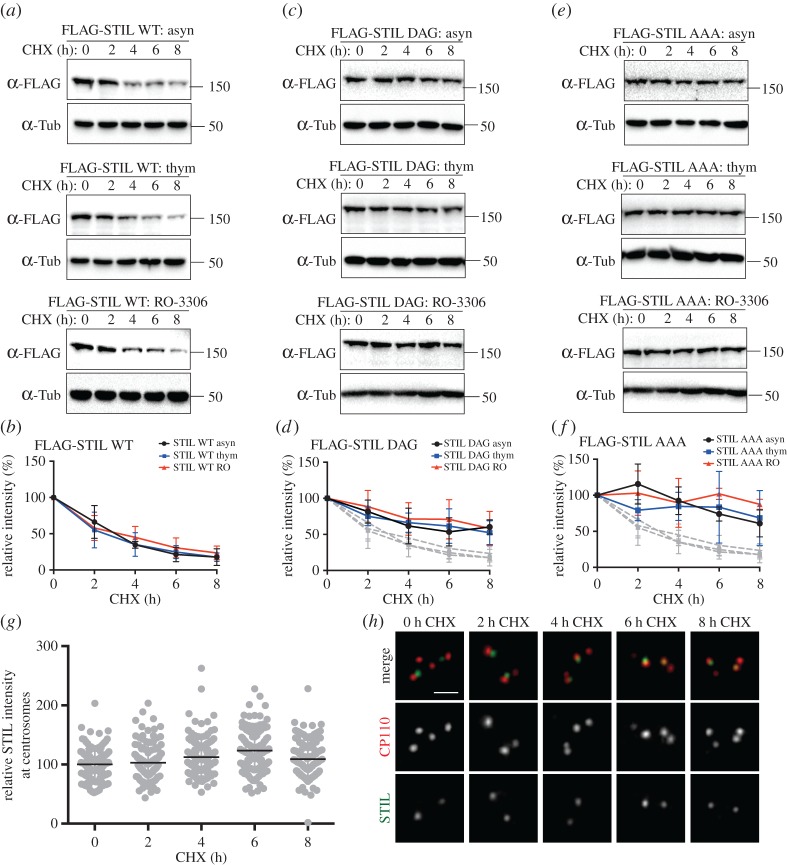
(*a*) U2OS cells were transfected with FLAG-STIL WT for a total of 60 h and either left untreated (asyn, upper panel), synchronized in S phase by thymidine treatment (thym, middle panel) or arrested in G2 phase by RO-3306 treatment (RO-3306, lower panel). Cells were subsequently incubated with cycloheximide for a total of 8 h, and samples were taken every 2 h to check for FLAG-STIL protein levels by western blotting with anti-FLAG antibodies. α-tubulin was analysed for loading control. (*b*) Graph shows quantification of relative FLAG-STIL WT intensity (normalized to time 0), as measured by western blotting in three independent experiments (as described in (*a*)). Curves for asynchronously growing cells are shown in black (asyn), for S-phase-arrested cells in blue (thym) and for G2-phase-arrested cells in red (RO). (*c*) The same experiment as described in (*a*), except that FLAG-STIL DAG was used for transfection. (*d*) The same analysis as in (*b*), except that graph shows quantification of relative FLAG-STIL DAG intensity. The grey curves present a repeat of (*b*), for comparison. (*e*) The same experiment as described in (*a*), except that FLAG-STIL AAA was used for transfection. (*f*) The same analysis as in (*b*), except that graph shows quantification of relative FLAG-STIL AAA intensity. The grey curves present a repeat of (*b*), for comparison. (*g*) U2OS cells were synchronized in S phase (24 h thymidine) before cycloheximide was added for up to 8 h. Cells were fixed every 2 h and stained with antibodies against STIL and CP110 (to identify centrioles). Graph depicts relative STIL intensity at each time point (normalized to time 0), as determined by immunofluorescence microscopy; data are compiled from two independent experiments, resulting in the analysis of 120 centrioles per time point. (*h*) Representative images of the experiment described in (*g*). STIL is depicted in green; CP110 in red. Scale bar indicates 1 µm.

Next, we sought to obtain temporal and spatial information about STIL degradation. First, we asked whether SCF-dependent STIL degradation occurs at any specific stage of the cell cycle, and with what kinetics. To this end, U2OS cells expressing either FLAG-STIL WT or FLAG-STIL DSG mutants were arrested at different cell cycle stages, before protein synthesis was blocked by addition of cycloheximide (CHX) and levels of FLAG-STIL proteins were monitored for 8 consecutive hours by western blotting. We found that exogenous STIL WT was degraded with similar kinetics, regardless of whether cells were growing asynchronously, arrested in S phase with thymidine or in G2 phase with RO-3306; in all cases, STIL levels dropped by approximately 75% after 8 h of CHX treatment (figure 3*a*,*b*). From these results, we conclude that STIL is subject to turnover throughout interphase of the cell cycle. By contrast, the transfected DSG mutants—STIL DAG (figure 3*c*,*d*) or STIL AAA (figure 3*e*,*f*)—showed markedly increased stability under all conditions, confirming that the observed decays in STIL levels were largely due to SCF-mediated degradation via the DSG motif. To obtain spatial information about STIL degradation, we next used immunofluorescence microscopy to examine levels of endogenous, centrosome-associated STIL. Although 24 h thymidine arrest caused some centrosome amplification in U2OS cells, we focused quantitative analyses on the majority of cells showing normal centrosome numbers. We found that STIL levels at the centrosomes of U2OS cells remained remarkably stable, even after CHX treatment for up to 8 h (figure 3*g*,*h*). This suggests that the association of STIL with the centrosome probably protects the protein from recognition by βTrCP and subsequent proteasomal degradation, implying that degradation mostly affects the cytoplasmic pool of STIL. Furthermore, these data confirm that the bulk of STIL is stably incorporated into centrioles [27], restricting its exchange with the cytoplasmic pool.

### 2.4. Mutation of the STIL DSG motif causes centriole amplification

**Figure 4.**
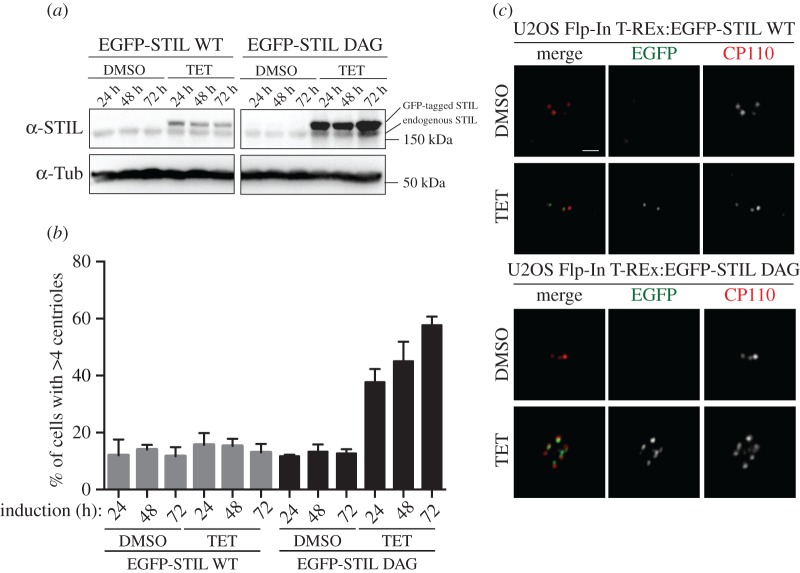
(*a*) U2OS Flp-In T-REx cells harbouring EGFP-STIL WT or EFGP-STIL DAG were treated for 24, 48 or 72 h with DMSO (for control) or tetracycline (TET) to induce transgene expression. Cell lysates were separated by SDS–PAGE and analysed by western blotting using antibodies against STIL or α-tubulin (loading control). Note that EGFP-tagged versions of STIL can readily be distinguished from endogenous STIL by their retarded migration on SDS–PAGE. A slight increase in the level of endogenous STIL might reflect stabilization or cleavage of the GFP-tag from the transgene product. (*b*) Cells were treated as described in (*a*) and then fixed and stained with antibodies against the centriole marker CP110, before centrioles were counted by immunofluorescence microscopy. Graph shows percentages of cells harbouring greater than four centrioles, depending on the indicated conditions. Three hundred cells were analysed for each condition (compiled from three independent experiments). (*c*) Representative images are shown of cells treated for 72 h with DMSO or tetracycline (TET), as described in (*a*) and analysed in (*b*). CP110 is shown in red, GFP in green. Scale bar indicates 1 µm.

Having established that SCF-βTrCP mediates cytoplasmic STIL turnover in interphase cells, we next asked whether interference with SCF-mediated STIL degradation would suffice to promote centriole amplification. Because transient transfection generally results in high levels of protein expression, so that even STIL WT already triggers substantial centrosome amplification [54], we used a Flp-In T-REx system, which allowed us to express WT and DAG mutant versions of EGFP-STIL in a tetracycline-inducible manner from the same genomic locus. In this system, induction of EGFP-STIL WT expression for up to 72 h resulted in protein levels that were only marginally above those seen for endogenous STIL (figure 4*a*), and this modest level of overexpression did not trigger centriole amplification (figure 4*b*,*c*), confirming previous results [39]. In stark contrast, 24–72 h after tetracycline addition, EGFP-STIL DAG was expressed to much higher levels than either EGFP-STIL WT or endogenous STIL, in line with the expectation that impaired degradation causes an accumulation of this mutant (figure 4*a*). Staining of the corresponding cells with anti-CP110 antibodies revealed that EGFP-STIL DAG triggered massive centriole amplification, with roughly 40% of cells containing greater than four centrioles already after 24 h of expression, and this phenotype was seen in nearly 60% of cells after 72 h (figure 4*b*,*c*). These results demonstrate that impaired SCF-mediated degradation of STIL results in sufficient STIL stabilization to trigger massive centriole amplification.

### 2.5. Inhibition of CDK2 activity triggers loss of STIL

**Figure 5.**
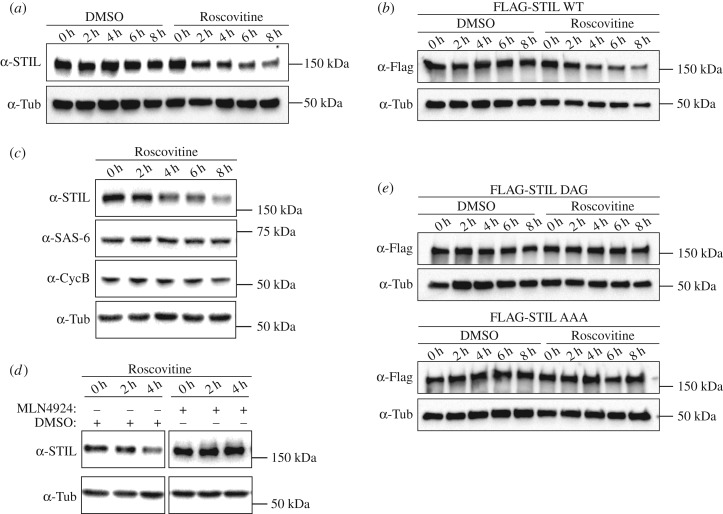
(*a*) U2OS cells were arrested in S phase with thymidine and treated with Roscovitine (or DMSO for control) for up to 8 h. Cell lysates were prepared every 2 h, separated by SDS–PAGE and analysed by western blotting using anti-STIL antibodies. α-tubulin was analysed as loading control. (*b*) HEK 293T cells were transfected with FLAG-STIL WT for a total of 72 h and arrested in S phase with thymidine, before addition of Roscovitine for up to 8 h (or DMSO for control). Cell lysates were prepared every 2 h, separated by SDS–PAGE and analysed by western blotting using anti-STIL antibodies. α-tubulin was analysed as loading control. (*c*) U2OS cells were arrested in S phase with thymidine and treated with Roscovitine for up to 8 h. Cell lysates were prepared every 2 h, separated by SDS–PAGE and analysed by western blotting using antibodies against STIL, SAS-6, Cyclin B or α-tubulin (loading control). (*d*) U2OS cells were arrested in S phase with thymidine and incubated with MLN4924 (or DMSO for control), before being treated with Roscovitine for up to 4 h. Cell lysates were prepared every 2 h, separated by SDS–PAGE and analysed by western blotting using antibodies against STIL or α-tubulin (loading control). (*e*) The same experiment as described in (*b*), except that FLAG-STIL DAG and FLAG-STIL AAA mutants were used for transfection.

One question raised by our discovery of SCF-mediated degradation of STIL relates to the identity of the kinase(s) involved in phosphorylation of S395 within the DSG motif. In an attempt to identify this upstream kinase(s), we have explored a possible involvement of several candidates. Specifically, we have added inhibitors of PLK4 (centrinone), PLK1 (BI-2536) and GSK-3 (GSK-3 inhibitor IX), as well as inhibitors with broader specificity (staurosporine, PKC-412) to S-phase-arrested U2OS cells and then monitored STIL levels by western blotting. Unfortunately, no reproducible stabilization of STIL could be observed under any of these conditions and so this assay failed to identify the kinase(s) responsible for phosphorylation of S395. However, in the course of these experiments, we discovered an unexpected link between STIL abundance and CDK2 activity. When adding the CDK inhibitor Roscovitine to S-phase-arrested U2OS cells, we were surprised to see a gradual decrease in STIL protein levels, amounting to a drop of roughly 60% after 8 h of treatment (figure 5*a*). Very similar results were obtained when using the CDK2-specific inhibitor SNS-032, which also caused a substantial drop in STIL protein levels after 8 h of treatment even at the lowest concentration assayed (0.1 mM) (electronic supplementary material, figure S5*a*). Together, these data indicate that CDK2 inhibition triggers a loss of STIL protein, which in turn implies that under physiological conditions CDK2 contributes to stabilize STIL.

To explore possible mechanisms accounting for the above observations, we asked whether CDK2 regulates STIL abundance at the transcriptional level. CDK2 has in fact been implicated in the regulation of E2F transcription factors at the G1/S phase transition [87,88] and STIL has been identified as a potential E2F target gene [89]. Thus, inhibition of CDK2 activity could potentially decrease the production of STIL mRNA. To explore this possibility, the Roscovitine time course experiment was repeated, but instead of monitoring endogenous STIL, we examined the fate of FLAG-STIL WT transiently transfected into HEK 293T cells. This ectopically expressed FLAG-STIL protein showed a very similar response to CDK2 inhibition as the endogenous STIL (figure 5*b*), even though it was expressed from a promoter that is not expected to respond to E2F. We thus conclude that the Roscovitine-induced loss of STIL is not caused by a transcriptional mechanism.

Considering that STIL is degraded by APC/C [39,54], we also explored the possibility that CDK2 inhibition might affect STIL levels via regulation of this ubiquitin ligase. This possibility was suggested by a report indicating that CDK2 inhibition in S-phase-arrested cells can lead to reactivation of APC/C under certain experimental conditions [90]. However, both Cyclin B and SAS-6, two prominent substrates of APC/C, remained stable in response to Roscovitine treatment, arguing that APC/C reactivation did not occur and thus cannot explain the observed drop of STIL levels (figure 5*c*).

Having eliminated two potential explanations for the observed drop in STIL levels in response to CDK2 inhibition, we next asked whether this phenotype requires SCF-mediated degradation. To this end, we pre-treated cells with MLN4924 to inhibit SCF E3 ubiquitin ligases, before performing Roscovitine time course experiments. Owing to synergistic toxicity of the two compounds, cells could only be monitored for up to 4 h. This limitation notwithstanding, we found that inhibition of functional SCF E3 ubiquitin ligases by MLN4924 prevented the drop in STIL levels that was triggered by Roscovitine in the DMSO-treated control cells (figure 5*d*; electronic supplementary material, figure *S5b*). This led us to postulate that the enhanced STIL degradation seen upon CDK2 inhibition reflects the fact that CDK2 normally protects STIL against SCF-βTrCP-mediated degradation. If this were the case, one would predict that mutation of the DSG motif should render STIL levels resistant to CDK2 inhibition. To test this prediction, we transfected FLAG-STIL DSG mutants into HEK 293T cells and examined the stability of the corresponding proteins in the presence of Roscovitine. Whereas FLAG-STIL WT was sensitive to Roscovitine treatment (figure 5*b*), both FLAG-STIL DAG and FLAG-STIL AAA completely resisted Roscovitine-induced STIL degradation (figure 5*e*; electronic supplementary material, figure S5*c*). We thus conclude that the loss of STIL triggered by CDK2 inhibition requires an intact DSG motif, implying that CDK2 antagonizes SCF-βTrCP-mediated degradation of STIL.

### 2.6. Degradation-resistant STIL does not circumvent the requirement for CDK2 in centriole duplication

CDK2 is known to be required for initiation of centriole duplication, but the molecular basis for this requirement has remained elusive [69–72]. The data presented above indicate that one important function of CDK2 consists in antagonizing SCF-mediated degradation of STIL, thereby stabilizing STIL for centriole biogenesis at the G1/S transition. This prompted the question of whether stabilization of STIL represents the one key role of CDK2 in centriole duplication, as opposed to CDK2 having multiple targets relevant to the process. In the former case, we reasoned that it should be possible to bypass the requirement for CDK2 by providing cells with non-degradable STIL. It has previously been shown that centriole amplification can be triggered by overexpression of either PLK4 or STIL [49,51,54–56]. However, while PLK4-induced centriole amplification does not occur in the absence of CDK2 activity [49], it has not previously been tested whether centriole amplification caused by excess STIL also requires CDK2. If the sole function of CDK2 was to increase STIL stability at the G1/S phase transition, then providing sufficient amounts of STIL should trigger centriole amplification regardless of the CDK2 activity status in the cell.

**Figure 6.**
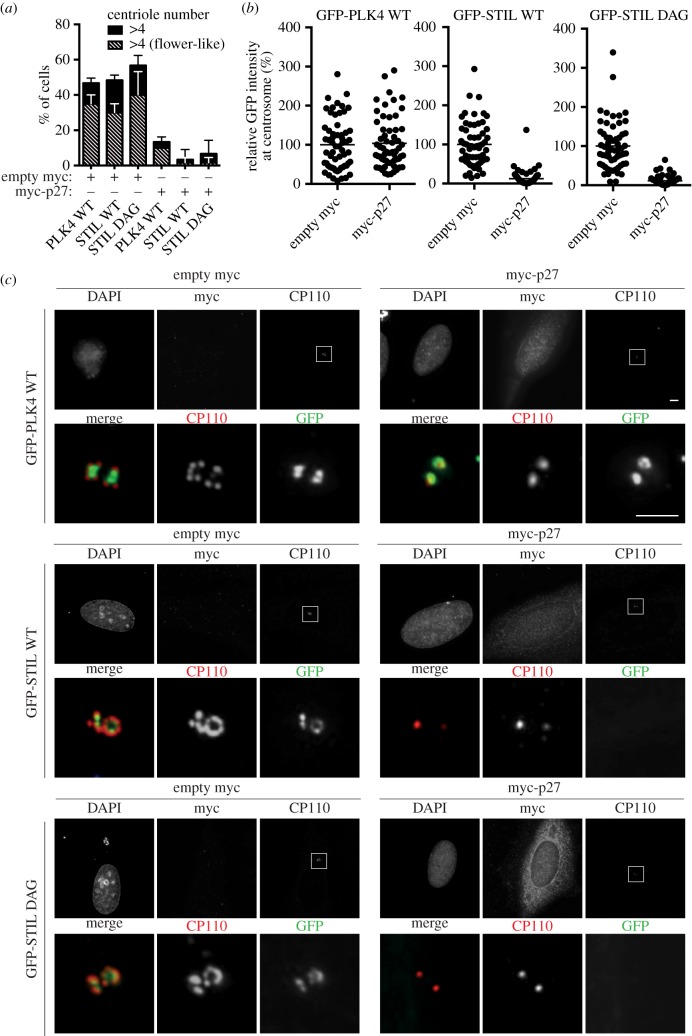
(*a*) U2OS cells were arrested in S phase with thymidine before being co-transfected for 36 h with the CDK2 inhibitor myc-p27 (or empty myc plasmid for control) and either GFP-PLK4 WT, GPF-STIL WT or GFP-STIL DAG. Cells were fixed and stained with anti-myc and anti-CP110 antibodies, and centrioles were counted by immunofluorescence microscopy, using myc and GFP signals to monitor successful co-transfection. The histogram shows percentages of cells with multiple centrioles (greater than 4, black bars), including the fraction displaying flower-like arrangements (hashed bars). A total of 60 cells were analysed for each condition (compiled from three independent experiments). Error bars denote s.d. (*b*) The centrosome-associated GFP signals representing GFP-PLK4 WT, GFP-STIL WT and GFP-STIL DAG (as described in (*a*)) were measured by fluorescence microscopy. Graphs represent the relative GFP intensities as observed after co-transfections of either myc-p27 or empty myc (control). Sixty centrosomes were analysed per condition (compiled from three independent experiments) and normalized to control (average set to 100%). (*c*) Representative images of the experiment described in (*a*). Upper panels show overviews of the entire cells, with DAPI, myc and CP110 stainings in grey, and centrosomes (CP110 dots) marked by white boxes. Scale bar indicates 4 µm. Lower panels show 10× magnifications of the boxed centrosomal regions, with CP110 shown in red and GFP in green. Scale bar indicates 2 µm.

To determine whether STIL stabilization might be sufficient to rescue centriole duplication in CDK2 inhibited cells, we co-transfected the CDK2 inhibitor myc-p27 (or empty myc plasmid for control) with EGFP-PLK4 or EGFP-STIL WT/DAG into S-phase-arrested U2OS cells. As expected, expression of PLK4, STIL WT or STIL DAG caused strong centriole amplification (approx. 50% of transfected cells harbouring greater than four centrioles) in the absence of CDK2 inhibition (empty myc plasmid) (figure 6*a*). By contrast, centriole amplification was blocked when PLK4 was co-expressed with myc-p27, as shown before [49], and, likewise, neither STIL WT nor STIL DAG were able to rescue centriole amplification in the presence of myc-p27 (figure 6*a*). This indicates that enhanced STIL stabilization is not sufficient to circumvent the requirement of CDK2 in centriole duplication, arguing that stabilization of STIL is not the sole function of CDK2 relevant to centriole biogenesis.

Regarding additional centriole-related function(s) of CDK2, we considered the possibility that CDK2 regulates not only the abundance of STIL but also its subcellular localization. In support of this notion, we observed that recruitment of STIL to centrioles was sensitive to the presence or absence of CDK2 activity. While overexpressed EGFP-PLK4 formed rings around pre-existing centrioles, regardless of CDK2 activity status and procentriole formation, both EGFP-STIL WT and EGFP-STIL DAG formed rings around pre-existing centrioles in control cells expressing active CDK2, but failed to accumulate at centrioles in cells harbouring myc-p27 (figure 6*b*,*c*). This suggests that CDK2 plays an important role not only in shielding STIL from SCF-βTrCP-mediated degradation, but also in controlling the recruitment of STIL to the sites of procentriole formation.

## 3. Discussion

**Figure 7.**
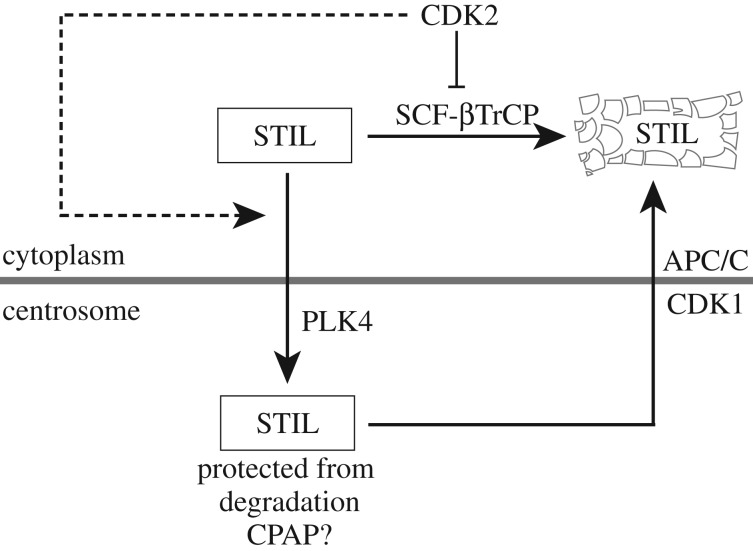
A proposed network controlling STIL abundance. According to this model, cytoplasmic STIL is degraded by SCF-βTrCP throughout interphase, while centrosome-associated STIL is protected, possibly through CPAP. CDK2 activity antagonizes SCF-βTrCP-mediated STIL degradation and additionally, may cooperate with PLK4 to enhance STIL recruitment to centrioles. During progression through M phase, the combined action of CDK1 and APC/C then results in STIL release from centrioles and cytoplasmic degradation, respectively [39].

It is well established that PLK4, STIL and SAS-6 cooperate to initiate the first steps in centriole biogenesis, and that the abundance of all three proteins is regulated by ubiquitin-dependent degradation [14,15,20]. While SCF-βTrCP controls levels of the kinase PLK4 [62–65], APC/C functions to degrade both STIL and SAS-6 during late M and early G1 phase [39,52,54,56,91]. Here, we describe a novel mechanism that contributes to centriole homeostasis in human cells by controlling the abundance and localization of the centriole duplication factor STIL. Through the use of quantitative mass spectrometry, we identified STIL as a direct target of SCF ubiquitin ligases. Moreover, we found that the F-box protein βTrCP binds a DSG motif located within the N-terminus of STIL, and that mutation of this degron leads to STIL stabilization and consequent centriole overduplication. We also discovered an unexpected link between STIL and CDK2. Our data suggest that CDK2 activity protects STIL against SCF-βTrCP-mediated degradation and, additionally, may contribute to control STIL recruitment to centrioles. Figure 7 summarizes these findings in a schematic model.

As substrate recognition by SCF-βTrCP generally depends on degron phosphorylation, we sought to identify the kinase acting on S395 within STIL's DSG motif. However, although we tested several kinase inhibitors for their ability to stabilize STIL, including inhibitors targeting plausible candidate kinases such as PLK4, PLK1 and GSK-3, we have been unable to identify the kinase(s) acting on S395. These negative results might reflect redundancies among multiple kinases, and therefore do not exclude a role for PLK4, PLK1, GSK-3 or any other kinase in the regulation of STIL abundance. Alternatively, however, it is also conceivable that phosphorylation of S395 may not be strictly required for STIL degradation, which would explain our inability to identify a pertinent kinase through stabilization-based assays. Some support for this possibility stems from the observation that S395 can be readily detected in cell lysates, which would not be expected if phosphorylation of this residue was the rate-limiting step triggering STIL degradation.

Collectively, our results make a strong case implicating SCF ligases in the regulation of STIL abundance. Moreover, they specifically link βTrCP to STIL degradation via a DSG degron. Surprisingly, however, siRNA-mediated depletion of βTrCP did not allow us to detect robust accumulation of STIL. Although this may reflect incomplete depletion of βTrCP, it also raises the possibility that other F-box proteins, or even other MLN4924-sensitive ligases, may cooperate with SCF-βTrCP to regulate STIL levels. Co-regulation of important regulatory proteins by multiple F-box proteins and cullin-RING ubiquitin ligases has previously been observed, as exemplified by Wee1 and Cdt1 [92–95].

Although the DSG motif within STIL is well conserved in most vertebrates, it is conspicuously absent in several rodent species, notably mouse and rat, indicating that subtle differences exist in the regulation of STIL levels in different organisms. For example, it is intriguing to consider the possibility that the physiological role of SCF-βTrCP-mediated degradation of vertebrate STIL may differ depending on the mode of centriole inheritance during fertilization. In humans and most mammals, the entry of a spermatozoon into an egg results in the introduction of a centriole, which can then be duplicated during each zygotic division [96,97]. By contrast, the first zygotic divisions in mouse and rat are acentriolar, before centrioles form *de novo* at the blastocyst stage [98–101]. It thus appears tempting to speculate that differences in STIL regulation between rodents and other mammals might somehow relate to differences in centriole inheritance and *de novo* biogenesis during early development.

Our data suggest that STIL is protected from SCF-βTrCP-mediated degradation as soon as this centriole duplication factor has been recruited to and integrated into centrioles. While future work will be required to address the mechanism underlying this protection, we note that STIL physically interacts with CPAP and that the STIL–CPAP interaction is essential for successful completion of centriole duplication [55,56,102,103]. It is intriguing, therefore, that the DSG motif within STIL is located in very close proximity to the purported CPAP binding motif (PRPXXP) [102,103]. Considering that the two motifs are only a few amino acids apart, this raises the possibility that CPAP binding to STIL results in masking of the DSG motif, thereby protecting STIL from degradation. If this were the case, our results would predict that STIL and CPAP interact primarily at centrioles, rather than within the cytoplasm.

Finally, we report the unanticipated observation that CDK2 activity interferes with SCF-βTrCP-mediated STIL degradation. The precise mechanism underlying this antagonism remains to be determined, but one possibility is that CDK2 directly phosphorylates STIL, thereby interfering with recognition of STIL by βTrCP. In this context, it is interesting that STIL was reported to bind the prolyl-isomerase PIN1 [104] and that phosphorylation-induced prolyl-isomerization has previously been shown to antagonize βTrCP-mediated protein degradation [105]. Alternatively, CDK2 might interfere with SCF-βTrCP activity itself. Although CDK2 activity has long been implicated in the regulation of centriole numbers [69–72,106], the precise centriole-related function(s) of this kinase remain to be elucidated. Our observation raised the possibility that one key function of CDK2 consists in stabilizing STIL at the G1/S transition. However, overexpression of either WT STIL or DSG mutant versions of STIL did not bypass the requirement for CDK2 activity for centriole overduplication, indicating that CDK2 must play additional roles. One prospect suggested by our data is that CDK2 regulates not only STIL abundance but also STIL recruitment to centrosomes. In fact, whereas centrosome-association of PLK4 was independent of the activity status of CDK2, overexpressed STIL did not localize to centrioles when CDK2 was inhibited.

As depicted schematically in figure 7, we propose that cytoplasmic STIL is subject to degradation by SCF-βTrCP throughout interphase. According to this model, SCF-βTrCP-mediated STIL degradation ensures that STIL levels do not rise above a critical threshold at inappropriate times, thus preventing unscheduled centriole (over-)duplication. At the G1/S phase transition, however, a rise in CDK2 activity interferes with this STIL degradation pathway. This then allows the accumulation of STIL to levels sufficient for centriole duplication. In addition, our data suggest that CDK2 is required for the recruitment of STIL to centrioles, where integration into a procentriolar structure is proposed to protect STIL from degradation. One attractive possibility is that this protection results from masking of the DSG degron by binding of CPAP to a neighbouring site on STIL. Upon passage through the next M phase, STIL will again be released into the cytoplasm, in response to phosphorylation by CDK1, where it is degraded by APC/C [39]. While additional work will be required to substantiate or refute the model depicted in figure 7, these findings propose new lines of investigation into the mechanisms that underlie the regulation of centriole duplication during cell cycle progression.

## 4. Material and methods

### 4.1. Cloning procedures

Site-specific point mutations were introduced into STIL WT via the QuikChange II XL Site-Directed Mutagenesis Kit (Agilent Technologies, Santa Clara, CA, USA), using the Gateway entry vector pENTR/D-TOPO_STIL 1-1287 [39] as template and oligonucleotides ccaatacatgatcacgacgctggtgttgaagatgaag and cttcatcttcaacaccagcgtcgtgatcatgtattgg to generate the point mutation S395A, and oligonucleotides gatgccaatacatgatcacgccgctgctgttgaagctgaagatttttctccaagac and gtcttggagaaaaatcttcagcttcaacagcagcggcgtgatcatgtattggcatc to generate the point mutations D394A, S395A and G396A, respectively. The Gateway LR Clonase Enzyme mix (Thermo Fisher Scientific, Waltham, MA, USA) was used to catalyse the recombination of entry vectors (pENTR) with destination vectors (pDEST) to generate expression vectors (pEXP). The entry vector pENTR/D-TOPO_STIL_S395A was recombined with destination vectors pDEST_pcDNA3.1_N3xFLAG [39] and pgLAP1 (Addgene plasmid number 19702, gift from Peter Jackson) to generate expression vectors pEXP_N3xFLAG_STIL_S395A (used to express FLAG-STIL S395A) and pEXP_NEGFP_S_FRT_STIL_S395A (used to express EGFP-STIL S395A and to generate U2OS Flp-In T-Rex cell line harbouring EGFP-STIL DAG). The entry vector STIL D394A, S395A, G396A was recombined with pDEST_pcDNA3.1_N3xFLAG [39] to generate expression vector pEXP_N3xFLAG_STIL_D394A, S395A, G396A (used to express FLAG-STIL AAA). The PLK4 full-length sequence was amplified from a myc-PLK4 plasmid [49] by polymerase chain reaction using oligonucleotides CACCGCGACCTGCATCGGG and TCAATGAAAATTAGGAGTCG. The blunt-ended PCR product was ligated into the pENTR/D- TOPO vector (Invitrogen, Carlsbad, CA, USA) to generate the entry vector PLK4 pENTR/D-TOPO_PLK4, which was subsequently recombined with the destination vector pgLAP1 by LR reaction to generate pEXP_NEGFP_S_FRT_PLK4 (used to express EGFP-PLK4). Clonings of myc-βTrCP1 (FBXW1A) [107], myc-p27 [71], pEXP_NEGFP_S_FRT_STIL fl [39] and FLAG-STIL FL, N-ter, MD, C-ter ΔC, ΔN [39] were described previously.

### 4.2. Cell culture, cell synchronization and transfections

All cells were grown in a 37°C incubator with 5% CO_2_ and regularly tested for mycoplasma. U2OS and HEK 293T cells were maintained in Dulbecco's modified Eagle's medium (DMEM), supplemented with 10% fetal calf serum (FCS) and 5% PenStrep (Thermo Fisher Scientific). The doxycycline-inducible U2OS T-REx cell line harbouring myc-tagged PLK4 [51] was cultured in the same medium, except that tetracycline-free fetal bovine serum was used and the medium was supplemented with 500 µg ml^−1^ G418 (Biovision, Milpitas, CA, USA) and 50 µg ml^−1^ hygromycine (Thermo Fisher Scientific). Expression of myc-PLK4 was induced by addition of 10 ng ml^−1^ doxycycline. Generation of the tetracycline-inducible U2OS Flp-In T-Rex cell line harbouring EGFP-tagged STIL WT has been described previously [39]. The tetracycline-inducible U2OS Flp-In T-REx cell line harbouring EGPF-STIL DAG was generated according to the manufacturer's protocols (Thermo Fisher Scientific). To select for transgene integration and culture cells after selection, DMEM with 10% tetracycline-free fetal bovine serum, 5% PenStrep (Thermo Fisher Scientific), 100 µg ml^−1^ hygromycin (Thermo Fisher Scientific) and 15 µg ml^−1^ blasticidine (Thermo Fisher Scientific) was used. Transgene expression was induced with 1 µg ml^−1^ of tetracycline. To synchronize cells in S phase, 2 mM thymidine (Sigma-Aldrich, St Louis, MO, USA) was applied for 24 h (except for the experiment described in figure 6, where thymidine was added for 36 h). To synchronize cells in G2 phase, 10 µM RO-3306 (Merck Millipore, Darmstad, DE, USA) was applied for 24 h. Transient transfections of 293T and U2OS cells were performed using TransIT-LT1 transfection reagent (Mirus Bio, Madison, WI, USA) according to the manufacturer's protocol.

### 4.3. Immunofluorescence microscopy

U2OS cells were fixed and permeabilized in methanol (5 min, −20°C). U2OS Flp-In T-REx cells were fixed in 3% paraformaldehyde for 15 min, followed by permeabilization with 0.5% Triton X-100 for 2 min. Fixed samples were prepared for immunofluorescence microscopy, as described previously [71], and analysed using a DeltaVision microscope on a Nikon TE200 base (Applied Precision, Issaquah, WA, USA) with a Plan Apochromat 60× 1.42 N.A. oil-immersion objective (Olympus, Tokyo, Japan) and 1.6× auxiliary magnification. A CoolSNAP HQ2 camera (Photometrics, Tucson, AZ, USA) was used to capture images. Serial optical sections acquired 0.2 µm apart along the *z*-axis were deconvoluted and projected into one image using Softworx (Applied Precision). Primary antibodies were rabbit anti-STIL (CA66 [54]), mouse anti-CP110 (91.390.21; EMD Millipore), rabbit anti-CP110 [108] and mouse anti-myc (9E10). Mouse and rabbit anti-CP110 antibodies were directly coupled to Alexa-555, and mouse anti-myc antibodies to Alexa-647, using antibody labelling kits (Thermo Fisher Scientific). Alexa-488-labelled anti-rabbit and Alexa-555-labelled anti-mouse secondary antibodies were from Thermo Fisher Scientific. For quantifications of STIL, GFP-PLK4 and GFP-STIL WT/DAG levels at centrosomes, ImageJ was used to measure intensities; background signal intensity was subtracted from cytoplasmic regions adjacent to centrosomes. Identical image acquisition and processing settings were applied whenever measurements were used for comparison (OMERO; Open Microscopy Environment [109]).

### 4.4. Cell extracts, immunoprecipitation and western blot analysis

Cells were lysed as described before [63], using either Tris (50 mM Tris–HCl, pH 7.4, 150 mM NaCl, 0.5% NP-40 (IGEPAL CA-630) or RIPA (50 mM Tris–HCl, pH8.0, 150 mM NaCl, 5 mM EDTA, 1% NP-40 (IGEPAL CA-630), 0.5% sodium deoxycholate (SDC), 0.1% SDS) lysis buffers, supplemented with protease and phosphatase inhibitors. For immunoprecipitation experiments, cell extracts (2–5 mg total protein) were incubated for 2 h at 4°C with Affi-Prep protein A matrix beads (Bio-Rad Laboratories, Hercules, CA, USA) cross-linked with 9E10 anti-myc antibodies. After incubation, beads were washed four to six times with wash buffer (50 mM Tris–HCl, pH 7.4, 150–300 mM NaCl, 0.5–1% IgePal), eluted with gel sample buffer and analysed by western blotting. Rabbit anti-STIL (ab89314; Abcam, Cambridge, UK), rabbit anti-Cep135 (ABE1857; EMD Millipore), mouse anti-Aurora A (610939; BD Biosciences, San Jose, CA, USA), mouse anti-FLAG (M2; Sigma-Aldrich), mouse anti-myc (9E10), mouse anti-SAS-6 (91.390.21; EMD Millipore), mouse anti-Cyclin B1 (GN53; Merck Millipore, Darmstad, DE, USA) and mouse anti-α-tubulin (T9026; Sigma-Aldrich) antibodies were used for western blotting. Western blots were developed using SuperSignal West Femto maximum sensitivity chemiluminescent reagent (Thermo Fisher Scientific) and a CCD-based imaging system (Bio-Rad, Hercules, CA, USA). ImageJ was used to measure band intensities and background signal intensity was subtracted.

### 4.5. Protein sequence alignments

Protein sequences were fetched using BLAST (Basic Local Alignment Search Tool) and aligned with the ClustalW algorithm in the program CLC Main Workbench 7 (v. 7.6.4 CLC Bio, Qiagen, Aarhus, Denmark).

### 4.6. Sample preparation for LC–MS analysis

To analyse phosphorylation sites on STIL, 3 × 10^6^ HEK 293 T cells were transfected for 48 h with FLAG-STIL FL [39], collected and lysed in 500 µl Tris lysis buffer (50 mM Tris–HCl, pH 7.4, 150 mM NaCl, 0.5% NP-40 (IGEPAL CA-630), supplemented with protease and phosphatase inhibitors. After centrifugation (15 min, 4°C, 16 000*g*), cell extracts were incubated at 4°C with Anti-FLAG M2 Affinity Gel (Sigma-Aldrich) for 2 h, thereafter beads were washed three times with Tris lysis buffer, followed by three washes with phosphate-buffered saline. Proteins were eluted with 100 mM glycine, pH 2.8, neutralized with 1 M Tris–HCl (pH 8.0), reduced in 5 mM Tris-2-carboxyethyl-phosphin (TCEP) for 10 min at 95°C, alkylated in 10 mM iodoacetamide at 25°C for 30 min in the dark and incubated in 12.5 mM *N*-acetylcysteine at 25°C for 10 min. Proteins were digested by incubation with sequencing-grade modified trypsin (1/50, w/w; Promega, Madison, WI, USA) overnight at 37°C and peptides were cleaned up using C18 Sep-Pak Vac columns (Waters, Baden-Dättwil, Switzerland) according to the manufacturer's instructions. Samples were dried under vacuum and stored at −80°C until further use.

To determine relative protein abundance, 4–6 × 10^6^ HEK 293T and 2 × 10^6^ U2OS T-REx cells were collected and lysed in 100 µl lysis buffer (1% SDC, 0.1 M ammoniumbicarbonate, 10 mM TCEP) using strong ultra-sonication (10 min, Bioruptor, Diagnode). Protein concentration was determined by BCA assay (Thermo Fisher Scientific) using a small aliquot. Sample aliquots containing 50 µg of total proteins were reduced for 10 min at 95°C and alkylated by adding chloroacetamide to 15 mM final concentration for 30 min at 37°C. Proteins were digested by incubation with sequencing-grade modified trypsin (1/50, w/w; Promega) overnight at 37°C. An aliquot of a heavy reference peptide mix containing 16 chemically synthesized proteotypic peptides (AQUA-grade, Thermo Fisher Scientific, see Bauer *et al*. [78] for details) was spiked into each sample at a concentration of 10 fmol of heavy reference peptides per 1 µg of total endogenous protein mass. Then, the peptides were cleaned up using iST cartridges (PreOmics, Munich) according to the manufacturer's instructions. Samples were dried under vacuum and stored at −80°C until further use.

### 4.7. Targeted PRM-LC-MS analysis of selected peptides/proteins

In a first step, parallel reaction monitoring (PRM) assays [110] were generated from a mixture containing 500 fmol of each heavy reference peptide and shotgun data-dependent acquisition (DDA) LC-MS/MS analysis on a Q-Exactive HF platform. The set-up of the μRPLC-MS system was as described previously [111]. Chromatographic separation of peptides was carried out using an EASY nano-LC 1000 system (Thermo Fisher Scientific), equipped with a heated RP-HPLC column (75 µm × 30 cm) packed in-house with 1.9 µm C18 resin (Reprosil-AQ Pur, Dr Maisch). Peptides were analysed per LC–MS/MS run using a linear gradient ranging from 95% solvent A (0.15% formic acid, 2% acetonitrile) and 5% solvent B (98% acetonitrile, 2% water, 0.15% formic acid) to 45% solvent B over 60 min at a flow rate of 200 nl min^−1^. Mass spectrometry analysis was performed on an Q-Exactive HF mass spectrometer equipped with a nanoelectrospray ion source (both Thermo Fisher Scientific). Each MS1 scan was followed by high-collision-dissociation of the 10 most abundant precursor ions with dynamic exclusion for 20 s. Total cycle time was approximately 1 s. For MS1, 3e6 ions were accumulated in the Orbitrap cell over a maximum time of 100 ms and scanned at a resolution of 120 000 FWHM (at 200 *m*/*z*). MS2 scans were acquired at a target setting of 1e5 ions, accumulation time of 50 ms and a resolution of 30 000 FWHM (at 200 *m*/*z*). Singly charged ions and ions with unassigned charge state were excluded from triggering MS2 events. The normalized collision energy was set to 27%, the mass isolation window was set to 1.4 *m*/*z* and one microscan was acquired for each spectrum. The acquired raw files were database searched against a human database (UniProt: download date: 4 February 2016, total of 20 204 entries) by MaxQuant software (v. 1.0.13.13) using default parameters. The best six transitions for each peptide were selected automatically using an in-house software tool and imported to Skyline (version 1.4). Two mass isolation lists containing the peptides of the medium and low abundant protein, respectively, were exported from Skyline and imported into the QE-HF operating software for PRM analysis using the following settings: the resolution of the orbitrap was set to 60/120 k FWHM (at 200 *m*/*z*) and the fill time was set to 150/250 ms to reach a target value of 3e6 ions for the medium/low-abundance peptides. Ion isolation window was set to 0.4 Th and the first mass was fixed to 100 Th. An MS1 scan using the same conditions as for DDA was included in each MS cycle. Each condition was analysed in biological triplicates. All raw files were imported into Skyline for protein/peptide quantification. To control for variation in injected sample amounts, the total ion chromatogram (only comprising ions with two or more charges) of each sample was determined using LFQ (see below) and used for normalization.

### 4.8. Quantitative shotgun LC–MS analysis

After five PRM LC–MS analysis, a standard DDA LC–MS analysis of the previous sample was carried out using the same gradient and MS parameters as described above. These samples were also included in the subsequent label-free quantification analysis to increase the number of identified and quantified proteins. The generated raw files were imported into the Progenesis QI software (Nonlinear Dynamics (Waters), v. 2.0) and analysed using the default parameter settings. MS/MS data were exported directly from Progenesis QI in mgf format and searched against a decoy database the forward and reverse sequences of the predicted proteome from *Homo sapiens* (download date: 4 February 2016, total of 41 158 entries) using MASCOT (v. 2.4.1). The search criteria were set as follows: full tryptic specificity was required (cleavage after lysine or arginine residues); three missed cleavages were allowed; carbamidomethylation (C) was set as fixed modification; oxidation (M) as variable modification. The mass tolerance was set to 10 ppm for precursor ions and 0.02 Da for fragment ions. Results from the database search were imported into Progenesis QI and the final peptide measurement list containing the peak areas of all identified peptides, respectively, was exported. This list was further processed and statically analysed using our in-house developed SafeQuant R script [111]. The peptide and protein false discovery rate was set to 1% using the number of reverse hits in the dataset. For the phosphorylation site analysis of antibody-enriched FLAG-STIL samples (preparation see above), we carried out standard DDA LC–MS analyses, using the same parameters as above, but adding phosphorylation (STY) as variable modifications for database searching.

### 4.9. Mass spectrometry: accession codes

The proteomics data have been deposited to the ProteomeXchange Consortium via the PRIDE [112] partner repository with the dataset identifier PXD008272 and 10.6019/PXD008272.

## Supplementary Material

Supplementary Figure 1;Supplementary Figure 2;Supplementary Figure 3;Supplementary Figure 4;Supplementary Figure 5

## Supplementary Material

Supplementary Figures Caption

## Data accessibility

This article has no additional data.
