# Supplementary material for: The SKP1-Cullin-F-box E3 ligase βTrCP and CDK2 cooperate to control STIL abundance and centriole number
Source: Open Biol. 2018 Feb 14;8(2):170253. doi: 10.1098/rsob.170253 (PMC5830536; doi:10.1098/rsob.170253)
Supplement: Supplementary Figures Caption [file rsob170253supp2.docx]

Supplementary Figure 1

1. U2OS cells were treated for 24 hours with 0.1 or 0.5 μM MLN4924 (or DMSO for control), before they were fixed and stained with antibodies against CP110 and STIL and centrioles (CP110 dots) were counted by immunofluorescence microscopy. The histogram shows percentages of cells with multiple centrioles (>4, black bars), including the fraction displaying flower-like arrangements (hashed bars). A total of 300 cells were analysed (compiled from 3 independent experiments). Error bars denote SD.
2. Representative images for the DMSO (control) and 0.5 μM MLN4924 conditions, used for counting of centrioles as described in A. CP110 is shown in red, STIL in green. Scale bar marks 1 μm.
3. Scatter plots show relative protein levels of Aurora A, Beta-Catenin and ORC1 upon treatment of HEK 293T cells with either DMSO (control, n=3) or 0.5 μM MLN4924 (n=3), as determined by label-free mass spectrometry. To indicate fold-changes in protein levels in response to MLN4924-treatment, the average values measured in DMSO-treated control cells were set to 1.0. Error bars denote SD, p-values from t-tests (two tailed, unpaired) are indicated.
4. Same as C, except that relative protein levels of tubulin alpha, tubulin beta and glycerinaldehyd-3-phosphat-dehydrogenase (GAPDH) are shown.
5. Scatter plots show relative protein levels of the indicated centriolar proteins upon treatment of S phase (thymidine) arrested HEK 293T cells with either DMSO (control, n=3) or 0.5 μM MLN4924 (n=3), as determined by PRM mass spectrometry. To indicate fold-changes in protein levels in response to MLN4924-treatment, the average values measured in DMSO-treated control cells were set to 1.0. Error bars denote SD, p-values from t-tests (two tailed, unpaired) are indicated.

Supplementary Figure 2

1. U2OS T-Rex cells harbouring myc-PLK4 were arrested in S phase (thymidine) and treated for 24 hours with doxycycline to induce expression of myc-PLK4 (10 ng/mL) or without doxycycline (0 ng/mL) for control. Cell lysates were separated by SDS-PAGE and analysed by Western blotting using anti-myc antibodies to detect myc-PLK4. α-tubulin was analysed as loading control.
2. Cells treated as described in A were fixed and stained with antibodies against CP110, and centrioles were counted by immunofluorescence microscopy. The histogram shows percentages of cells with multiple centrioles (>4, black bars), including the fraction displaying flower-like arrangements (hashed bars). A total of 300 cells were analysed (compiled from 3 independent experiments). Error bars denote SD.
3. Scatter plot shows relative STIL protein levels after treating cells as described in A, followed by mass spectrometric analysis of cell lysates by PRM. To indicate fold-changes in protein levels in response to MLN4924-treatment, the average values measured in DMSO-treated control cells were set to 1.0. Error bars denote SD, p-values from t-tests (two tailed, unpaired) are indicated.

Supplementary Figure 3:

Multiple protein sequence alignment (ClustalW) of several rodent STIL protein sequences, including human STIL shown on top. Note that the DSG motif is not conserved in most rodent species.

Supplementary Figure 4:

Annotated tandem mass spectrum showing identification of the peptide phosphorylated on serine 395. FLAG-STIL was overexpressed in HEK 293T cells and concentrated from cell lysate by immunoprecipitation using anti-FLAG antibodies prior to mass spectrometry analysis. The Mascot ion score and delta score are illustrated to show identification and localization confidence, respectively.

Supplementary Figure 5:

1. S phase (thymidine) arrested U2OS cells were treated for 8 hours with DMSO (for control) or the indicated concentrations of the CDK2 selective inhibitor SNS-032 (0.1, 0.5, 1 mM) or 50 mM Roscovitine (for comparison). Cell lysates were separated by SDS-PAGE and subjected to Western blot analysis using anti-STIL antibodies. α-tubulin was analysed as loading control.
2. Graph shows relative STIL intensities upon co-treatment of U2OS cells with MLN4924 (or DMSO for control) and Roscovitine for 0, 2 or 4 hours, as determined by Western blotting. Data are compiled from 4 independent experiments, one of which is shown in Figure 5D. STIL intensities for the control conditions (0 hour Roscovitine treatment) were set to 100%. Error bars denote SD.
3. Graph shows quantification of relative FLAG-STIL WT, FLAG-STIL DAG and FLAG-STIL AAA intensities upon Roscovitine treatment of S phase arrested HEK 293T cells for either 0 (control), 2, 4, 6 or 8 hours. Intensities were measured by Western blotting in 3 independent experiments, one of which is illustrated in Figures 5B (STIL WT intensity) and E (STIL DAG and AAA intensities). STIL intensities were set to 100% in the control condition (0 hour Roscovitine treatment) and normalized against loading control (alpha-tubulin signal). Error bars denote SD.
